# Cost-Effective Electrochemical Activation of Graphitic Carbon Nitride on the Glassy Carbon Electrode Surface for Selective Determination of Serotonin

**DOI:** 10.3390/s20216083

**Published:** 2020-10-26

**Authors:** Vijayaraj Kathiresan, Thenmozhi Rajarathinam, Seulah Lee, Suhkmann Kim, Jaewon Lee, Dinakaran Thirumalai, Seung-Cheol Chang

**Affiliations:** 1 Graduate Department of Chemical Materials, Pusan National University, Busan 46241, Korea; vijaya.raj6@gmail.com; 2 Department of Cogno-Mechatronics Engineering, Department of Optics and Mechatronics Engineering, College of Nanoscience and Nanotechnology, Pusan National University, Busan 46241, Korea; thenmozhi@pusan.ac.kr; 3 College of Pharmacy, Pusan National University, Busan 46241, Korea; leeseulah@pusan.ac.kr (S.L.); neuron@pusan.ac.kr (J.L.); 4 Department of Chemistry, Pusan National University, Busan 46241, Korea; suhkmann@pusan.ac.kr

**Keywords:** electrochemical activation, serotonin sensor, graphitic carbon nitride nanosheets

## Abstract

A simple one-step electrochemical deposition/activation of graphitic carbon nitride (g-C_3_N_4_) is highly desired for sensor configurations and remains a great challenge. Herein, we attempt an electrochemical route to exfoliate the g-C_3_N_4_ nanosheets in an aqueous solution of pH 7.0 for constructing a sensor, which is highly sensitive for the detection of serotonin (5-HT). The significance of our design is to exfoliate the g-C_3_N_4_ nanosheets, a strong electrocatalyst for 5-HT detection. Investigations regarding the effect of neutral pH (pH 7.0) on the bulk g-C_3_N_4_ and g-C_3_N_4_ nanosheets, physical characterization, and electrochemical studies were extensively carried out. We demonstrate that the g-C_3_N_4_ nanosheets have a significant electrocatalytic effect for the 5-HT detection in a dynamic linear range from 500 pM to 1000 nM (*R*^2^ = 0.999). The limit of detection and sensitivity of the designed 5-HT sensor was calculated to be 150 pM and 1.03 µA µM^−1^ cm^−2^, respectively. The proposed sensor has great advantages such as high sensitivity, good selectivity, reproducibility, and stability. The constructed g-C_3_N_4_ nanosheets-based sensor platform opens new feasibilities for the determination of 5-HT even at the picomolar/nanomolar concentration range.

## 1. Introduction

Serotonin (5-hydroxytryptamine, 5-HT), a redox-active neurotransmitter, is one of the monoamine neurotransmitters prevailing in the mammalian central nervous system. Serotonin is directly associated with the body for regulation of mood, sexuality, emesis, pain, sleep, and appetite [1]. The fluctuations of 5-HT transmission are linked to several neurological disorders, e.g., multiple sclerosis, neuroblastoma, and Parkinson’s disease [2]. The physiological levels of 5-HT is ~15 nM in the human body fluids, and the altered levels can cause brain-related diseases. Therefore, it is crucial to rapidly detect 5-HT for the clinical diagnosis [1,3]. Among a plenty of conventional analytical techniques, the electrochemical system is highly favorable for clinical diagnosis due to prominent features such as reliability, affordability, portable, high selectivity, and good sensitivity without demanding any sophisticated process. Regardless of the above-mentioned advantages, the electrochemical approach for selective detection of 5-HT in practical applications is quite limited due to the interference with similar electroactive species (e.g., ascorbic acid). To achieve the goal of selective 5-HT detection, an effective strategy to negotiate the interfering species is of utmost importance.

In recent years, most of the literature studies have focused on the electrochemical sensors utilizing heavy-metal complexes along with the toxic material analysis for improving the charge transfer and fast signaling response [4,5]. To avoid the metal toxicity introducing the cost-effective carbon-based nanomaterials sensor are preferable and could also serve as the biocompatible substrate for further ex vivo and in vivo investigations [6]. To date, a lot of research works are reported on the electrochemical production of graphene-based nanomaterials and their electrochemical properties [7,8,9]. The electrochemically constructed high-purity monolayer structure of graphene is quite compatible with the design of sensors and many biomolecules could be readily detected in real samples as well. In our previous literature, we proposed the fabrication of a nanocomposite sensor for the ex vivo detection of dopamine neurotransmitters using brain tissue samples of a mouse Parkinson’s disease model [10].

The design and fabrication of a metal-free carbon nanomaterials sensor, especially graphitic carbon nitride (g-C_3_N_4_) with a two-dimensional (2D) layered structure, is an analogue of graphene. The intriguing physicochemical and electrochemical properties of the g-C_3_N_4_ shows great versatility for applications in catalysts, lithium-ion batteries, hydrogen, optoelectronic devices, and electrochemical sensors [11,12,13,14,15]. In this context, the synthesis of high-quality monolayer g-C_3_N_4_ nanosheets with reduced structural defects became very crucial, and many researchers are focused on this issue. Ultrathin g-C_3_N_4_ nanosheets have recently been synthesized by prolonged ultra-sonication using a bulk g-C_3_N_4_ liquid exfoliation technique. Yet, this process might enable the preparation of a single-layered g-C_3_N_4_ structure, desirable for photocatalytic applications [16]. Cheng et al. [17] carried out chemical exfoliation of g-C_3_N_4_ sheets with sulfuric acid (H_2_SO_4_) as a strong oxidizing agent. However, other literature has shown that ultrathin g-C_3_N_4_ nanosheets developed by an electrophoretic method using the mixture of NaOH and melamine [18]. This method requires high voltage and alkaline medium with the complicated synthetic procedure, which proves very tedious and yields only negligible quantities. All these reports reinforce that the g-C_3_N_4_ nanosheets are more suitable electrochemical catalysts for sensor applications. Jiang et al. [19] pioneered their research on electrochemical sensors using bulk g-C_3_N_4_ to detect DA neurotransmitters. Gao et al. [20] also reported on the electrochemical g-C_3_N_4_ nanosheets sensor for the determination of cadmium. The porous g-C_3_N_4_-based composite was used for ascorbic acid detection [21]. The above reports consist of complex synthesis procedures and often urge researchers to focus on an easy electrochemical exfoliation of g-C_3_N_4_ nanosheets. Electrochemical exfoliation, which requires no costly equipment and is faster, more effective, and more controllable and is more appropriate to exfoliate g-C_3_N_4_ nanosheets from bulk g-C_3_N_4_ [22,23]. Moreover, there is no reported literature on the electrochemical determination of 5-HT using the g-C_3_N_4_ nanosheets.

**Scheme 1:**
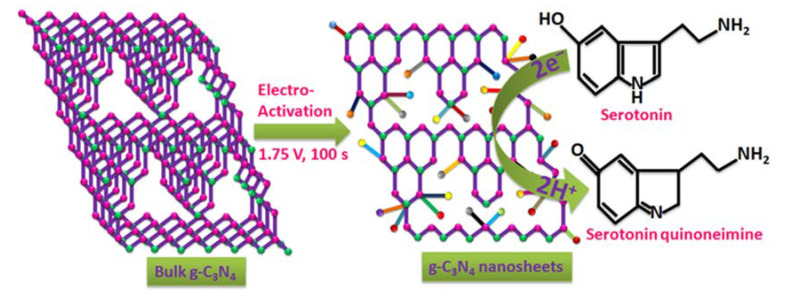
Schematic representation of a one-step electrochemical activation of g-C_3_N_4_ nanosheets for 5-HT detection.

Our aim is to develop cost-effective g-C_3_N_4_ nanosheets by simple electrochemical activation method. To our knowledge, this is the first report for the electrochemical 5-HT detection using g-C_3_N_4_ nanosheets (Scheme 1).

Initially, the bulk g-C_3_N_4_ was drop-casted onto the glassy carbon electrode (GCE) surface and electrochemically activated at the applied potential of 1.75 V in neutral pH conditions (pH 7.0) to exfoliate g-C_3_N_4_ nanosheets from the bulk g-C_3_N_4_. The sensor has been optimized by varying pH and applied potential. The high electro-catalytic activity of the g-C_3_N_4_ nanosheets enhances the sensitivity and selectivity of the 5-HT sensors.

## 2. Materials and Methods

### 2.1. Reagents and Instruments

Melamine, serotonin (5-HT), tyrosine (Tyr), glucose (GLU), ascorbic acid (AA), uric acid (UA), sodium phosphate dibasic (Na_2_HPO_4_), sodium phosphate monobasic (NaH_2_PO_4_), sodium hydroxide (NaOH), and phosphoric acid (H_3_PO_4_) were purchased from Sigma Aldrich, USA. The 5-HT solution was freshly prepared each day and protected from light during use.

All chemicals were of analytical grade and were used as received. An amount of 0.1 M phosphate buffer solution (PBS) at different pH values was prepared by mixing a solution of Na_2_HPO_4_ and NaH_2_PO_4_ at different proportions, the pH adjusted with NaOH or H_3_PO_4_. All aqueous solutions were prepared using deionized water (Milli-Q water purifying system, 18 MΩ·cm).

Electrochemical experiments including cyclic voltammetry (CV) and chronoamperometry were carried out with a CompactStat potentiostat (Ivium Technologies B.V., De Zaale 11, 5612 AJ Eindhoven, The Netherlands) employing a conventional three-electrode system: a bare glassy carbon electrode (GCE, 3 mm in diameter) as the working electrode, Ag/AgCl as the reference electrode, and a platinum wire as the auxiliary electrode. The bulk g-C_3_N_4_ was electrochemically activated on indium tin oxide (ITO) substrates to study their surface characteristics by a field-emission scanning electron microscopy (FE-SEM, Hitachi S-4200, Chiyoda, Japan) at 15 kV and 150 W.

### 2.2. Sensor Preparation

The bulk g-C_3_N_4_ was synthesized through the direct heating process, as reported previously [24]. Briefly, 10 g of melamine was heated at 550 °C for 2 h under air atmosphere. The resultant yellow powders were then washed with deionized water and ethanol several times. Finally, the powers were dried at 60 °C in air overnight. The as-obtained bulk g-C_3_N_4_ was dispersed in water (3 mg mL^−1^) and sonicated for 2 h. A bare GCE was polished to using 0.05 µm alumina powder on a polishing cloth with water rinsing and dried at room temperature. Some 5 µL of 3 mg mL^−1^ bulk g-C_3_N_4_ suspensions were drop-casted on the GCE surface and allowed to dry in ambient temperature (25 °C) conditions. The bulk g-C_3_N_4_ modified GCE was subjected to a constant potential of 1.75 V for 100 s (optimal) in aqueous solution (pH 7.0) to get the g-C_3_N_4_ nanosheets. The prepared sensor was thoroughly rinsed with deionized water followed by drying in air. Finally, the sensor was represented as g-C_3_N_4_ nanosheets and was stored at 4 °C in the refrigerator when not in use. Moreover, an additional 5 µL of 3 mg mL^−1^ bulk g-C_3_N_4_ suspensions were drop-casted on the GCE surface and allowed to dry in ambient temperature (25 °C) conditions and used as a bulk g-C_3_N_4_-modified GCE.

## 3. Results and Discussion

### 3.1. Physical Characterization of g-C_3_N_4_ Nanosheets

**Figure 1:**
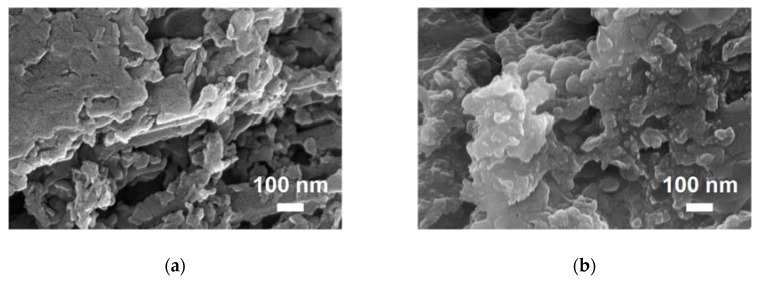
FE-SEM images of (**a**) bulk g-C_3_N_4_ and (**b**) electrochemically activated g-C_3_N_4_ nanosheets.

The surface morphology of bulk g-C_3_N_4_ and the electrochemically activated g-C_3_N_4_ nanosheets characterized by FE-SEM are shown in Figure 1.

The bulk g-C_3_N_4_ possesses a folded grain-like morphology with numerous agglomerates forming a thick layer, similar to the previous reports of the other researchers [16]. After electrochemical activation, the g-C_3_N_4_ nanosheets revealed a porous 2D-like network with a large surface area. The g-C_3_N_4_ nanosheets show a sponge-like morphology due to the heavily smashed surfaces, which indicates the successful exfoliation of bulk g-C_3_N_4_ to g-C_3_N_4_ nanosheets.

### 3.2. Optimization of g-C_3_N_4_ Nanosheets Synthesis

**Figure 2:**
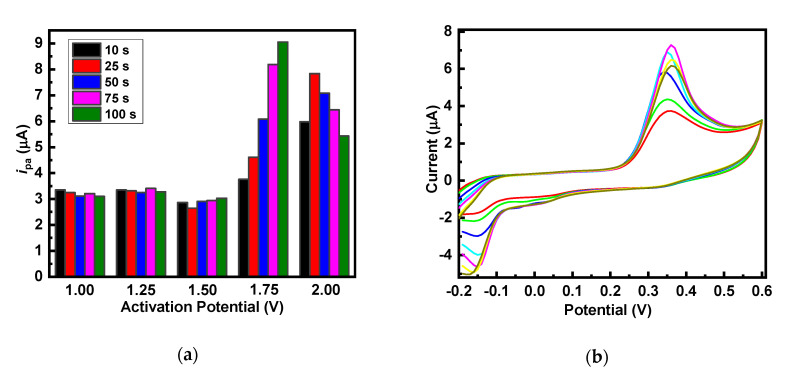

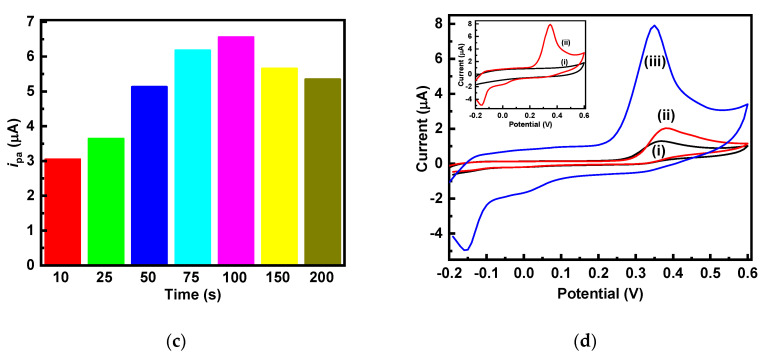
(**a**) Amperometric current response of 5-HT oxidation using various applied potential against different times. (**b**) CVs of g-C_3_N_4_ nanosheets were carried out at a constant applied potential of 1.75 V against different times. (**c**) The corresponding current response of 5-HT oxidation against different times. (**d**) CVs of (i) bare GCE, (ii) bulk g-C_3_N_4_, and (iii) g-C_3_N_4_ nanosheets in 0.1 M PBS (pH 7.0) containing 30 µM 5-HT at a scan rate of 50 mVs^−1^. Inset: CV curves of g-C_3_N_4_ nanosheets in the (i) absence and (ii) presence of 30 µM 5-HT.

Herein, we attempted a simple electrochemical approach to exfoliate g-C_3_N_4_ nanosheets from the bulk g-C_3_N_4_ in aqueous solution (pH 7.0) without any surfactants. The effect of various applied potential against time in seconds was investigated by the chronoamperometric experiments. The optimal applied potential that could exfoliate the bulk g-C_3_N_4_ into g-C_3_N_4_ nanosheets was determined to be 1.75 V for 100 s, as shown in Figure 2a.

The significant enhancement in the electrochemical sensing performance reveals a well-differentiated very sharp oxidation peak that remains constant at various potentials and at various times, as shown in Figure 2b and c. The strong electrocatalytic properties of the formed g-C_3_N_4_ nanosheets are thin enough to penetrate electrons [25]. These results were similar to the electrochemical activation of carbon-based nanomaterials [26,27]. Therefore, the constant potential of 1.75 V for 100 s is ideal to enable further electrochemical measurements with the g-C_3_N_4_ nanosheets electrodes.

The electrochemical performance of 5-HT on bare GCE (i), bulk g-C_3_N_4_ (ii), and g-C_3_N_4_ nanosheets (iii) modified electrodes were evaluated by CV experiments in PBS (pH 7.0) containing 30 µM 5-HT at the scan rate of 50 mVs^−1^, and the results are shown in Figure 2d. The bare GCE and the bulk g-C_3_N_4_ shows irreversible oxidation for 5-HT with anodic peak current of 1.08 µA and 1.81 µA at 0.37 V, respectively. While, the g-C_3_N_4_ nanosheets modified GCE shows a quasi-reversible redox behavior for 5-HT with anodic peak current of 8.26 µA at 0.35 V and cathodic peak current of −5.45 µA at −0.15 V. The g-C_3_N_4_ nanosheets modified GCE display an increased current response, and the peak potential shift to less positive as compared to the bulk g-C_3_N_4_. The electrode oxidation reaction involves a two-electron process, which is accompanied by a transfer of two protons, forming 5-HT quinoneimine. The new reduction peak observed only for the g-C_3_N_4_ nanosheets (−0.15 V) is most likely due to the reduction in the quinone group on 5-HT quinoneimine [28]. The reduction reaction, which occurs within the chosen potential window is due to the fast electron transfer property of the g-C_3_N_4_ nanosheets. The electrochemically active surface areas were also calculated by using the Randles–Sevcik equation (Equation (1)).

*i*_pa_ = 2.69 × 10^5^*n*^3/2^*ACD*^1/2^*ν*^1/2^ (1)

where, *i*_pa_ is the anodic peak current (A), *n* is the number of electrons (*n* = 2), *A* is the electrochemically active surface area (cm^2^), *D* is the diffusion coefficient (9.0154 × 10^−6^ cm^2^ s^−1^), *C* is the concentration of 5-HT (30 × 10^−6^ M), and *ν* is the scan rate (V s^−1^). According to this equation, the electrochemically active surface area of the bare GCE, bulk g-C_3_N_4_, and g-C_3_N_4_ nanosheets modified electrodes were calculated to be 0.071 cm^2^, 0.118 cm^2^, and 0.539 cm^2^, respectively. The g-C_3_N_4_ nanosheets modified electrodes possess higher electroactive surface area, which enhances the oxidation of 5-HT. The feasibility the g-C_3_N_4_ nanosheets modified GCE towards the catalytic oxidation of 5-HT was examined in the absence and presence of 5-HT in PBS. In the inset Figure 2d, the g-C_3_N_4_ nanosheets show no redox peak in the absence of 5-HT, and in the presence of 30 µM 5-HT, a redox peak was observed, due to the electrocatalytic activity towards the 5-HT. This result indicates that the g-C_3_N_4_ nanosheets modified GCE in 5-HT produced 3.5 and 6.5 times more current response (6.64 µA) than the bulk g-C_3_N_4_ (1.85 µA) and the bare GCE (1.10 µA). Therefore, the g-C_3_N_4_ nanosheets modified GCE could serve as a beneficial nanomaterial for sensitive 5-HT detection.

### 3.3. Optimization of 5-HT Sensors

**Figure 3:**
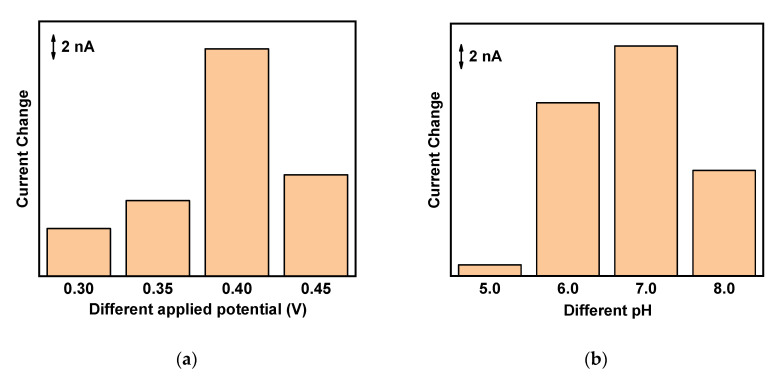
(**a**) Current change under different applied potential for the g-C_3_N_4_ nanosheets in 0.1 M PBS (pH 7.0) with the addition of 100 nM 5-HT. (**b**) Current change under different pH values for the g-C_3_N_4_ nanosheets in 0.1 M PBS with the addition of 100 nM 5-HT at an applied potential of 0.40 V.

In order to determine the optimum conditions, chronoamperometric measurements under various applied potential, and at various pH levels, were explored. From the potential range from 0.30 V to 0.45 V along with the addition of 100 nM 5-HT in 0.1 M PBS (pH 7.0) was evaluated and the results are shown in Figure 3a. 

Figure 3a reveals the highest current response at 0.40 V. The optimum pH for the 5-HT sensor was measured in the pH range from 5.0 to 8.0 with the addition of 100 nM 5-HT at an applied potential of 0.40 V and are depicted in Figure 3b. Figure 3b shows maximum current responses for the sensor in PBS pH 7.0. Therefore, the optimized applied potential of 0.40 V and pH of 7.0 were chosen for the following experiments.

### 3.4. Calibration Plots

**Figure 4:**
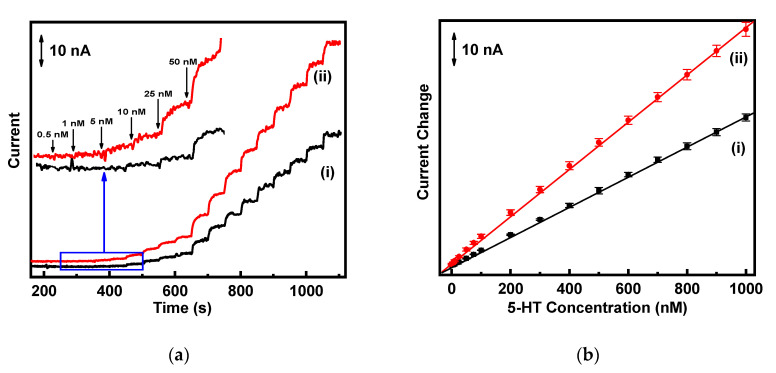
(**a**) Amperometric response of (i) bulk g-C_3_N_4_ and (ii) g-C_3_N_4_ nanosheets at 0.40 V in 0.1 M PBS (pH 7.0) with successive addition of 5-HT. Inset: The concentrations of 5-HT (0.5 nM, 1 nM, 5 nM, 10 nM, 25 nM, and 50 nM); (**b**) the corresponding calibration curves.

The analytical performance and calibration plots for bulk g-C_3_N_4_ and g-C_3_N_4_ nanosheets towards 5-HT detection are presented in Figure 4.

The amperometric measurements were carried out to examine the constructed sensor upon sequential addition of 5-HT aliquots in 0.1 M PBS (pH 7.0), at an operating potential of 0.4 V. After each addition of 5-HT, the current response increased within five seconds (Figure 4a). The 5-HT calibration curves were constructed; each error bar shown on the curve represents one standard deviation over five measurements, and the values are expressed in 95% confidence intervals. In the calibration curve (Figure 4b), the sensor demonstrates a dynamic linear range from 500 pM to 1000 nM, and the linear regression equation for g-C_3_N_4_ nanosheets is *i* (nA) = 0.476 *C*_5-HT_ (nM) + 0.072 (*R*^2^ = 0.999). The limit of detection for g-C_3_N_4_ nanosheets was calculated to be 150 pM (S/N = 3), the sensitivity was estimated to be 1.03 µA µM^−1^ cm^−2^, the limit of detection of the bulk g-C_3_N_4_ was 1.88 nM, and the sensitivity was 0.63 µA µM^−1^ cm^−2^. The sensitivity of g-C_3_N_4_ nanosheets is approximately 1.6 times higher than the bulk g-C_3_N_4_. The results were compared with studies reported previously and are given in Table 1.

Our g-C_3_N_4_ nanosheet results exhibit better limit of detection, and dynamic linear range and sensitivity is comparable with the studies reported previously. Therefore, these results suggest that the g-C_3_N_4_ nanosheets demonstrate better electrocatalytic activity towards 5-HT detection.

### 3.5. Selectivity Study

**Figure 5:**
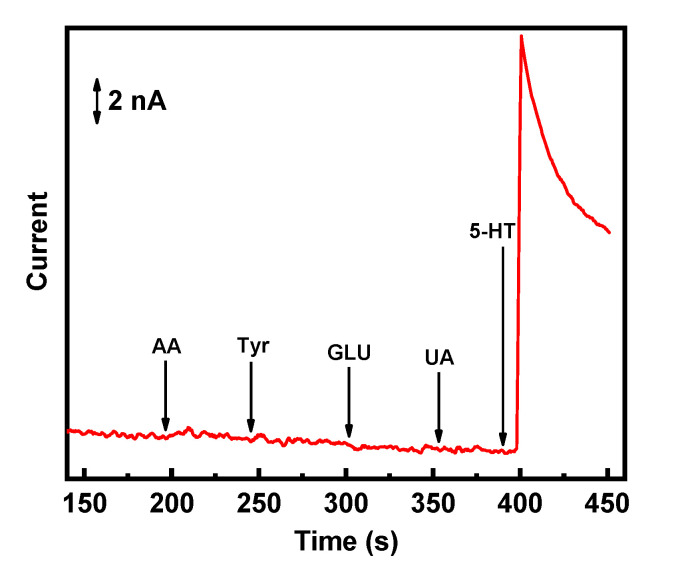
Amperometric response of g-C_3_N_4_ nanosheets for the addition of 2 µM AA, 2 µM Tyr, 2 µM GLU, 2 µM UA, and 0.2 µM 5-HT in 0.1 M PBS (pH 7.0) at an applied potential of 0.4 V.

Poor selectivity is a critical issue when the sensors are applied to real biological samples for 5-HT detection. The electrochemically synthesized carbon-based nanomaterials are likely to have a stronger electrocatalytic effect than the noble metals [9,27]. Therefore, the selectivity of the sensor was explored to study the interference towards 5-HT detection at the potential of 0.4 V. Figure 5 displays the amperometric measurements recorded on the g-C_3_N_4_ nanosheets electrode for the successive additions of 2 µM of AA, UA, Tyr, GLU, and finally, 0.2 µM of 5-HT.

The designed sensor shows a rapid and sharp increase in the current response for 5-HT, and there were no distinct current changes or interferences by other substances. The g-C_3_N_4_ nanosheets favorably enhance electrostatic interaction only with positively charged 5-HT and readily creates an electrostatic repulsion for AA (negatively charged at physiological pH) [32,33]. This result indicates that the g-C_3_N_4_ nanosheets can be favorable for practical sensor applications.

### 3.6. Reproducibility and Stability

Reproducibility and storage stability are important characteristics for 5-HT detection, and the designed sensor was examined initially and after 30 days through the amperometric experiments. The reproducibility of the four g-C_3_N_4_ nanosheets electrodes was recorded from the current response towards 100 nM 5-HT, and the relative standard deviation (RSD) was determined to be 5.08%. The storage stability of the sensor was measured by the current response of 100 nM 5-HT. After 30 days, the decrease in current response was sustained over 95% of its initial current response value. The proposed serotonin sensor showed an acceptable reproducibility and long-term storage stability for the detection of 5-HT.

## 4. Conclusions

A cost-effective and a simple one-step electrochemical activation of g-C_3_N_4_ nanosheets from bulk g-C_3_N_4_ in aqueous solution (pH 7.0) was carried out to fabricate the 5-HT sensor. The g-C_3_N_4_ nanosheets modified GCE possesses a high electroactive surface area, which ensures fast electron transfer. When compared with bulk g-C_3_N_4_, the electrochemically activated g-C_3_N_4_ nanosheets exhibit a strong electrocatalytic effect, which improves the sensitivity and selectivity towards 5-HT. In addition, the proposed sensor demonstrates good dynamic linear range, fast response, and picomolar detection limit. The prominent characteristics of g-C_3_N_4_ nanosheets open new opportunities for the easy fabrication of portable electrochemical sensors. Therefore, the designed sensor is a promising candidate for the ex vivo and in vivo 5-HT sensing applications.

## Figures and Tables

**Table 1:** Comparison of the prepared sensor with previously reported studies for 5-HT detection.

Modified Electrode	Method	Limit of Detection (nM)	Dynamic Linear Range (nM)	Sensitivity (µA µM^−1^ cm^−2^)	Ref.
AuAg-GR ^1^	Amperometry	1.6	2.7–4820	0.766	[1]
RGO-Co_3_O_4_^2^	DPV ^5^	48.7	100–51,000	2.2	[29]
MCM-41-COOH/Au@nano-CILPE ^3^	SWV ^6^	100	200–20,000	-	[30]
Fe_3_O_4_–MWCNT–poly(BCG)/GCE ^4^	DPV	80	500–100,000	-	[31]
GCE/g-C_3_N_4_ nanosheets	Amperometry	0.15	0.5–1000	1.03	This study

^1^ AuAg-GR: graphene-encapsulated AuAg alloy; ^2^ RGO-Co_3_O_4_: reduced graphene oxide-cobalt oxide; ^3^ MCM-41-COOH/Au@nano-CILPE: carboxyl-functionalized mesoporous molecular sieve/colloidal gold modified nano-carbon ionic liquid paste electrode; ^4^ Fe_3_O_4_–MWCNT–poly(BCG)/GCE: iron oxide–multiwalled carbon nanotubes–poly(bromocresol green); ^5^ DPV: differential pulse voltammetry; ^6^ SWV: square pulse voltammetry.
